# Lower prevalence of hip fractures in foreign-born individuals than in Swedish-born individuals during the period 1987-1999

**DOI:** 10.1186/1471-2474-11-203

**Published:** 2010-09-10

**Authors:** Björn Albin, Katarina Hjelm, Sölve Elmståhl

**Affiliations:** 1 School of Health Sciences and Social Work, Växjö University, and Department of Health Sciences, Division of Geriatric Medicine, Lund University, Sweden; 2 Department of Health Sciences, Division of Geriatric Medicine, Lund University, Sweden

## Abstract

**Background:**

This is the first longitudinal study with a 22-year follow-up, based on a national and complete sample, to determine whether the prevalence of hip fracture and the age when it occurs are influenced by migration and by being foreign-born. Cultural background and environmental factors such as UV-radiation and lifestyle during childhood and adolescence may influence the risk of a hip fracture event later in life. Differences in prevalence might occur between the indigenous population and those who have migrated to a country.

**Methods:**

The study was based on national population data. The study population consisted of 321,407 Swedish-born and 307,174 foreign-born persons living in Sweden during the period 1987-1999.

**Results:**

Foreign-born individuals had a reduced risk of hip fracture, with odds ratios (ORs) of 0.47-0.77 for men and 0.42-0.88 for women. Foreign-born women had the hip fracture event at a higher age on average, but a longer time spent in Sweden was associated with a small but significant increase in risk.

**Conclusions:**

We found that there was a reduced risk of hip fracture in all foreign-born individuals, and that the hip fracture event generally happened at a higher age in foreign-born women. Migration must therefore be considered in relation to the prevalence and risk of hip fracture. Migration can therefore have a positive effect on one aspect of the health of a population, and can influence and lower the total cost of healthcare due to reduced risk and prevalence of hip fracture.

## Background

The relation between the prevalence of hip fracture and migration is not well understood, and has not been studied longitudinally. Improvement in our knowledge is important in this respect since cultural background and physical environment during childhood and adolescence are known to be able to influence the risk of a hip fracture event [1], and differences in the prevalence of hip fracture might occur between the indigenous population and people who have migrated to a country.

Hip fractures are one of the most common reasons for elderly people being admitted to hospital, they are an important cause of death and disability, and measured by frequency and economic costs, they are a large public health problem [2].

Hip fractures are an increasing problem, since they are strongly associated with old age and the number of elderly people is increasing worldwide. In a global projection, it was estimated that the numbers will double between 1995 (1.3 million) and 2025 (2.6 million) [3]. The numbers of hip fractures vary in different parts of the world, with the greatest number in Europe. At present, about half of all hip fractures occur in Europe and North America [4,5]. Sweden has one of the highest prevalences of hip fractures in the world, especially among elderly [6].

Swedish society has changed during the last century due to international migration. In 1950, about 200,000 foreign-born people lived in Sweden, constituting 2.8 percent of the total population, as compared to 13 per cent (or slightly more than 1 million) in 2006 [7]. The population of foreign-born individuals in Sweden is a great mixture of different nationalities, but is dominated by labour migrants from the Nordic countries, especially Finland, and from other European countries such as former Yugoslavia, Germany, and Poland [7].

Hip fracture events could be related to osteoporosis or other systemic diseases, medication, medical history, anthropometric variables, lifestyle, or demographic variables such as age and sex [2]. Gender has a strong association with hip fractures and most cases, between 64% [8] and 75% [6], have been reported in women. Parental history may be of importance; studies have shown a modest but increased risk of hip fracture, in both men and women, if one of the the parents has had an osteoporotic fracture [9].

The influence of ethnicity, race, and different cultural background on the risk and prevalence of hip fracture has been studied to some extent. Hip fractures and the risk of hip fractures among the Japanese population in Hawaii has been investigated in several studies. The results have shown that there is a lower incidence and lower risk of falls and hip fractures in [Japanese people both in Japan and in Hawaii than in caucasians in Hawaii or on the mainland of the United States [10-13]. The incidence of hip fractures has been compared between different countries in Asia, and between Hong Kong and the UK [14-16]. The result showed that there is a higher incidence in more urbanized countries. A study in California showed a lower risk of hip fracture in individuals who were classified as black, latino, or Asian over a 5-year period, but non-English speakers had higher rates of hip fractures [17].

There have been two previous Swedish cross-sectional studies restricted to urban areas, one of which focused on hip fracture and migration. In a study of hip fractures in the elderly in Stockholm county, the significance of country of birth was investigated. Non-Scandinavian Europeans and women from Asia had a significantly lower incidence of hip fracture. Women from other Nordic countries, with the exception of Swedes, had a numerically but not statistically significantly lower incidence [6]. In another study, a trend break in the earlier increase in hip fracture incidence was found during the period 1992-1995. A possible explanation of the finding was the increasing numbers of non-Scandinavian immigrants living in the city of Stockholm [18].

There have been no longitudinal studies on the possible influence of migration and of being a foreign-born person on the risk and prevalence of hip fracture.

The aim of this study was to investigate whether prevalence of hip fracture and age when it occurs are influenced by migration and by being born in a foreign country.

## Methods

This longitudinal study was based on national population data. The total population of Sweden in 1970 was 8,081,142 and the study population consisted of 628,581 individuals, 321,407 of whom were Swedish-born and 307,174 of whom were foreign-born persons living in Sweden. The original database was set up by CAFO (the Centre for Labour Market Research) at Växjö University. The data originated from Statistics Sweden (SCB) and the Swedish National Board of Health and Welfare Centre for Epidemiology, covering the period 1970-1999 and including all 361,974 foreign-born individuals aged 16 years or more who were registered as living in Sweden in 1970. In relation to sex the group of foreign-born persons comprised of 168 702 men and 193 272 women. Thus there were more females then men. In addition, 361,974 matched Swedish controls were added.

The matching criteria were age (± 3 years), sex, occupation, type of employment, and living in the same county in 1970. Three groups were used to describe the type of employer: government, municipal, or other employer. County meant any one of 25 different geographical areas of Sweden and occupation was coded according to the Nordic Occupation Classification System (NYK).

All data originated from the National Census of 1970, which was a total census relating to the situation on November 1, 1970. By using data (up to December 31, 1999) from the National Population Register (RTB), a cross-check was performed and each person was assigned a code according to whether they were deceased, were still living in Sweden, or had emigrated--or if no information was available. Information from the National Board of Health and Welfare Centre for Epidemiology on cause of death and hospital admissions was added to the database. This Swedish national register of hospital care and diagnosis, which is run according to the International Classification of Diseases (ICD system), covers the period from 1987 to the present.

Subjects were excluded if they had migrated, if they had died before 1987, or if information from the following census in 1990 was missing. Thus, 95,367 individuals in total (13.2%) were excluded from the original database. As a consequence of the exclusion criteria 30 247 Swedish born and 16 014 foreign-born persons did not have a matched control person but 291 160 foreign-born had their matched Swedish control. In total the used database consisted of 307, 174 foreign-born and 321,407 Swedish born persons.

Those with occurrence of hip fracture were identified by the ICD codes S720-S722 and 820 according to ICD versions 9 and 10 [19,20], and time to first hip fracture event was used in the statistical analysis.

### Statistical analysis

To start with, the analysis involved a comparison between the total group of foreign-born individuals and the group of Swedish-born individuals regarding the number of hip fracture events. Analysis was also made in relation to sex and country/region of birth. Natives from the following countries were studied in particular: Denmark, Finland, Norway/Iceland, Yugoslavia, Poland, Germany, other European countries, and non-European countries. The rationale for studying these particular countries was that increased mortality had been shown in these migrant groups in previous investigations [21], and that they constituted the main groups (74.9%) of all migrants in Sweden that were included in the database during the period under study.

Values are given as numbers, means, and percentages. Hip fracture risks were calculated as odds ratios (ORs) with 95% confidence interval (CI). Comparisons were made by tests of significance with the Chi-squared test. A value of p < 0.05 was considered statistically significant [22]. The importance of being foreign-born and of country of birth adjusted for age on the dependent variable (hip fracture) was tested with logistic regression analysis. A separate analysis was done for those considered to have the highest risk of hip fracture, i.e. those who had reached the age of 70 years or more during the study period (1987-1999). Cox regression analysis, adjusted for age, was performed and included 14,884 individuals who had emigrated during the period 1987-1999.

All analyses were performed using SPSS software version 16.0.

### Ethics

At the time of this study all Swedish Universities had regional ethic committees and since national data were used approval from both the Ethics Committee of Lund University and all other local ethics committees in Sweden was obtained.

## Results

The study population with the two groups of 307,174 foreign-born and 321,407 Swedish-born subjects had a mean age in 1970 of 37.5 years and 37.9 years, respectively. The percentage of women was significantly higher in the foreign-born group, 54.3% versus 53.4% (p < 0.001).

A hip fracture event was found for 9,607 of the foreign-born individuals and for 12,452 of the Swedish-born individuals. The majority of those in both groups with a hip fracture event were women, 7,251 of the 9,607 foreign-born subjects (75%) and 8,897 of the 12,452 subjects who were Swedish-born (71.5%).

Mean age at study entry (1970) was noted. Foreign-born men with a hip fracture had a mean age of 49.1 years (95% CI = 48.6, 49.6) while Swedish-born men had a mean age of 49.7 years (95% CI = 49.3, 50.0). The corresponding mean ages for women were 55.2 years (95% CI = 54.9, 55.4) and 54.4 years (95% CI = 54.2, 54.7).

The distribution of country of birth among foreign -born persons with a hip fracture event differed. Of all patients who had had a hip fracture event, Finland (26.6%) and other European countries (20.5%) were most common as the country of birth for men, and Finland (28.7%) and Iceland/Norway (21.3%) were most common for women.

### The risk of hip fracture

The risk of having a hip fracture event was significantly higher for Swedish-born individuals (OR = 1.11, 95% CI = 1.095, 1.121) than for foreign-born individuals (OR = 0.87, 95% CI = 0.874, 0.901). Furthermore, Cox analysis adjusted for age also showed that for foreign-born subjects there was a longer time before the hip fracture event than for Swedish-born subjects. When analyses were performed separately for men and women in relation to country of birth, men had significantly higher risk if their country of birth was Norway/Iceland or if they were stateless, or if their country of birth was not known. For males born in Poland, Germany, or another European country, the risk was significantly lower, although their mean age in 1970 was higher than in men born in Sweden; see Table 1.

**Table 1:** Characteristics of male subjects regarding country of birth, mean age, and incidence of hip fracture^1^

Country of birth	Total number	Mean age in 1970	Hip fracture (n)	Hip fracture /10,000 person years	OR	p-value	Mean no. of years in Sweden in 1970
Sweden	150,107	37.0	3,555	20.4			

Denmark	12,753	39.9	305	21.9	1.01	0.868	11.8
Finland	52,317	32.7	627	10.5	0.58	< 0.001	12.9
Iceland/Norway	10,153	41.6	366	32.1	1.49	< 0.001	21.2
Yugoslavia	10,966	31.2	62	4.6	0.25	< 0.001	4.8
Poland	3,354	41.2	60	16.5	0.76	0.029	15.7
Germany	11,870	37.6	207	15.4	0.75	< 0.001	24.1
Other European country	28,354	38.3	484	15.2	0.75	< 0.001	20.9
Non-European country	10,641	39.4	235	20.4	0.94	0.294	31.5
Stateless/Unknown	121	41.0	10	82.6	3.71	< 0.001	31.0

^1^Adjusted for age

Women showed a similar pattern, with significantly higher risk for women born in Norway/Iceland, or a non-European country, or if they were stateless, or if their country of birth was unknown. A significantly lower risk but at the same time a higher mean age in 1970 was found for women born in Poland or Germany; see Table 2.

**Table 2:** Characteristics of female subjects regarding country of birth, mean age, and incidence of hip fracture

Country of birth^1^	Total number	Mean age in 1970	Hip fracture (n)	Hip fracture /10,000 person years	OR	p-value	Mean no. of years in Sweden in 1970
Sweden	171,300	38.9	8,897	44.4			

Denmark	11,733	40.7	633	47.7	1.04	0.343	22.7
Finland	74,770	35.3	2,079	23.6	0.61	< 0.001	18.8
Iceland/Norway	18,198	44.0	1,544	73.8	1.60	< 0.001	25.5
Yugoslavia	8,056	30.3	73	7.4	0.17	< 0.001	5.6
Poland	4,144	41.4	174	37.5	0.80	0.004	18.8
Germany	16,616	41.1	768	40.2	0.89	0.001	25.6
Other European country	23,430	41.9	1,209	44.4	0.99	0.827	24.3
Non-European country	9,529	45.3	753	75.2	1.52	< 0.001	34.2
Stateless/Unknown	169	48.6	18	106.5	2.17	0.001	20.6

^1^Sweden reference group

### Age and hip fracture

The mean age when the hip fracture occurred was similar (p = 0.174) in foreign-born men (73.8 years, 95% CI = 72.5, 73.5) and Swedish-born men (73.4 years, 95% CI = 73.0, 73.8).

Foreign-born women had a significantly higher mean age at their hip fracture event (78.9 years, 95% CI = 78.7, 79.2) than Swedish-born women (78.2 years, 95% CI = 77.9, 78.4) (p < 0.001).

A separate analysis was performed on those subjects who were considered to have the highest risk, those who would have become 70 years of age or more during the time period 1987-1999. No significant differences in mean age were found between foreign-born and Swedish- born men or women in this age group in relation to the time when the hip fracture occurred.

### Being foreign-born and hip fracture

Logistic regression analysis was performed to determine the importance of being foreign-born, with adjustment for age on the dependent variable hip fracture. Both age and being foreign-born were significant factors (p < 0.001); age had a OR = 1.10 and the corresponding OR for being foreign born was 0.82. The importance of being foreign-born and of country of birth, with adjustment for age on the dependent variable hip fracture, was also tested with logistic regression analysis, separately for men and women; see Tables 3 and 4.

**Table 3:** Multifactorial influence of country of birth on age-adjusted hip fracture in men, with Sweden as reference

Country of birth	B		S.E.		p-value		OR	
	Age	Ethnicity	Age	Ethnicity	Age	Ethnicity	Age	Ethnicity
Denmark	0.082	-0.139	0.001	0.061	< 0.001	0.023	1.09	0.87
Finland	0.081	-0.268	0.001	0.045	< 0.001	< 0.001	1.09	0.77
Iceland/Norway	0.081	0.120	0.001	0.057	< 0.001	0.035	1.09	1.13
Yugoslavia	0.082	-0.757	0.001	0.130	< 0.001	< 0.001	1.09	0.47
Poland	0.082	-0.647	0.001	0.133	< 0.001	< 0.001	1.09	0.52
Germany	0.083	-0.421	0.001	0.074	< 0.001	< 0.001	1.09	0.66
Other European country	0.083	-0.467	0.001	0.050	< 0.001	< 0.001	1.09	0.63
Non-European country	0.082	-0.366	0.001	0.070	< 0.001	< 0.001	1.09	0.69

Stateless/Unknown	0.082	0.879	0.001	0.348	< 0.001	0.012	1.09	2.41

**Table 4:** Multifactorial influence of country of birth on age-adjusted hip fracture in women, with Sweden as reference

Country of birth	B		S.E		p-value		OR	
	Age	Ethnicity	Age	Ethnicity	Age	Ethnicity	Age	Ethnicity
Denmark	0.092	-0.023	0.001	0.044	< 0.001	0.605	1.10	0.98
Finland	0.095	-0.290	0.001	0.026	< 0.001	< 0.001	1.10	0.76
Iceland/Norway	0.090	-0.211	0.001	0.030	< 0.001	< 0.001	1.09	0.81
Yugoslavia	0.092	-0.878	0.001	0.120	< 0.001	< 0.001	1.10	0.42
Poland	0.092	-0.405	0.001	0.081	< 0.001	< 0.001	1.10	0.67
Germany	0.093	-0.273	0.001	0.041	< 0.001	< 0.001	1.10	0.76
Other European country	0.093	-0.299	0.001	0.033	< 0.001	< 0.001	1.10	0.74
Non-European country	0.091	-0.132	0.001	0.042	< 0.001	0.002	1.10	0.88

Stateless/Unknown	0.092	-0.106	0.001	0.269	< 0.001	0.694	1.10	0.90

Being foreign-born was an independent risk factor, with lower risk (OR = 0.47-0.77) of causing hip fractures in men from all countries/regions of birth except Iceland/Norway (OR = 1.13), and in individuals who were stateless or whose country of birth was unknown. Countries/regions of birth were also a significant independent risk factor for hip fracture in women, except for women born in Denmark. Except for Denmark the risk was lower (OR = 0.42-0.88) in all other groups.

The importance of how long the foreign-born person had lived in Sweden, adjusted for age, was also analyzed with logistic regression analysis. Time in Sweden adjusted for age was a significant factor (p < 0.001, B-value = 0.005, annual OR = 1.005). When men and women were analyzed separately, time in Sweden was significant for women (p < 0.001, B-value = 0.005, annual OR = 1.01) but not for men (p = 0.230, B-value = 0.003, annual OR = 1.00).

The group with 16, 014 foreign-born persons without a Swedish twin had a significant higher mean age (54.3 vs. 37.5 years), a higher percentage of women (58.9% vs. 54.3%) and a significant higher percentage (10.1% vs. 3.1%) of fracture events than the foreign-born persons with matched control.

## Discussion

This is the first study with a 22-year follow-up, based on a national and complete sample, to investigate whether prevalence of and age when hip fracture occurs is influenced by migration and by being foreign-born. The main findings in the study were that after adjustment for age, foreign-born individuals living in Sweden had a reduced risk of hip fracture (OR = 0.82). Foreign-born women generally sustained a hip fracture at a higher age than Swedish-born women; the difference in age was 0.7 years. In foreign-born women, a longer time spent in Sweden (adjusted for age) was associated with a small but significantly increased risk of hip fracture.

Previous studies have shown an increased mortality risk and a different pattern of morbidity in foreign-born Swedes than in native Swedes [21,23]. The lower risk of hip fractures in foreign-born individuals in Sweden contrasts with earlier findings of health outcomes in this group. The result does, however, correspond to the only earlier Swedish cross-sectional study of hip fractures in migrants in Stockholm County, which was therefore restricted to an urban area. Our study is also in agreement with studies on hip fracture comparing European countries, which have shown a lower risk in Central Europe than in Northern Europe [3,24]. In earlier studies, country or region of origin and the environment during childhood and adolescence has been found to influence bone strength and to be of importance for the risk of hip fractures [1,17]. Even if migrants adapt to their new environment and to a new society, and acculturation takes place, the findings in this study stress the importance of the first period of life, during which the bone mass is established, in relation to the risk of hip fracture. There is also a possibility that when migrants enter a new society, some will still try to hold on to earlier preferences such as diet [25]. This could have an influence on the risk of hip fracture.

The result also corresponds to the finding that the general health of a migrant population has often been found to be better than that of the population of the country they migrate to. This has been explained as a "healthy migrant effect": migrants are a selection of the whole population and only people with good health migrate [26-28].

The increased risk of hip fracture found for foreign-born men and women from Iceland/Norway, for individuals who were stateless or had unknown country of birth, and for women from non-European countries can be partially explained by a higher mean age. For women, the increased risk is no longer evident when multifactorial analysis is performed, with adjustment for age. Thus, the increased risk is still evident for men when multifactorial analysis is performed. There is a high incidence of hip fracture in Norway as well as in Sweden. The high incidence in both countries may be due to similarities in diet, less exposure to ultraviolet radiation in the Nordic countries, or lifestyle [29-31]. The lower risk for men and women from Germany and Poland, and men from other European Countries, cannot be explained by differences in mean age but must be influenced by other factors related to country of birth and migration. Factors related to a person's early environment such nutrition, physical activity, and exposure to sun (influencing vitamin D metabolism), and genetic factors are known to be important [2,3,17]. However in this study we had no information about the individual's genetic poly morphology.

One factor that also could influence the demonstrated lower risk for hip fracture is an increased mortality among foreign-born persons. A higher mortality among migrants in Sweden have been shown in earlier studies (21, 23)

Another explanation might be the different situation for foreign-born people regarding their working environment. Earlier studies showed that foreign-born individuals were working in more manual and physically demanding occupations to a greater extent [32,33]. High levels of physical activity are known to reduce the risk of hip fracture [34,35].

Hip axis length has been shown to predict hip fracture but no data were available to study if this differs between Swedish and foreign-born persons in this study. However studies have found no differences in hip axis length in relation to ethnic background [36].

Body height and body fat composition could also influence the risk of hip fracture events and an earlier study have found differences between Swedes and foreign-born persons living in Sweden.

Foreign-born persons were found to be shorter (2.4 cm for women and 2.9 cm for men) than Swedes, have a higher Body Mass Index (BMI) and higher prevalence of obesity [37]. Lower height, higher BMI and obesity could reduce the risk of risk fracture event.

The age when hip fracture occurred was different (0.7 years later on average) only for foreign-born women, and this may have some importance from a public health point of view. This was not evident in the 70+ age group, where no differences were found. Fracture events before the age of 70 years occur later in foreign-born women, and this may be a indication of less osteoporosis due to the fact that peak bone mass was established before the age of 25 in a country with more sun exposure and higher bone mineral density (BMD) and less co-morbidity predisposing for falls and [36,38].

The logistic regression analysis showed that country/region of birth is an importance factor to consider in relation to risk of hip fracture. The lower risk of hip fracture in foreign-born individuals, except those from Norway/Iceland, can influence the prevalence in the total population. The percentage of foreign-born individuals has increased in Sweden, and today it is about 13% of the total population [7]. The decrease in hip fracture incidence reported from the city of Malmö could be the first sign of a change that is an effect of migration and of a larger percentage of foreign born persons in the Swedish population. This could have implications for the future in terms of healthcare planning and costs for healthcare and society in general. In 1996, hip fractures accounted for 332,332 days of care in hospital [37,39]. A total annual cost, including medical care, non-medical care, informal care, indirect costs, and value of quality adjusted life years lost, for fractures related to osteoporosis has been estimated to be 5,639 million Swedish crowns (600.3 million euros) in 2007 [38,40]. Migration and migrants are likely to contribute to reduced costs for fractures in Swedish society.

In foreign-born women, a longer time spent in Sweden was associated with a small but significantly increased risk of hip fracture (after adjustment for age). The result must be analyzed further, but could be an indication of adjustment to a new society [39,41].

Because of differences in cultural background and environment before and after migration, migration and the number of migrants in a population could influence the prevalence and risk of hip fractures. If an acculturation process and an adaptation take place [25] prevalence and risk could be adjusted to become more similar to the indigenous population as has been seen for some other diseases such as gastric carcinoma and diabetes [40,42,41,43]. If cultural values and habits are maintained after the migration, the prevalence and risk could be more similar to those in the migrant's country of origin.

The foreign group with no Swedish matched control could influence the result but since this group has a higher mean age, higher percentage of women and a higher percentage of fracture events the result with lower risk of hip fracture among foreign born persons are not overestimated but instead rather been underestimated.

## Conclusion

The main findings of this study were the reduced risk of hip fracture in foreign-born individuals, and the later time for hip fracture but increased risk of hip fracture in foreign-born women if they had spent a long time in Sweden. These results indicate that migration is an important factor to consider in relation to a possible reduction in the high hip fracture prevalence risk seen in the Scandinavian countries. Changes to a society in terms of a higher percentage of foreign-born individuals can have a positive effect on one aspect of the health of the population and can influence and even lower the total cost of care regarding hip fracture.

This study of the prevalence and risk of hip fracture in foreign-born individuals and native Swedes from 1987 to 1999 was based on national data from Statistics Sweden and from the Swedish National Board of Health and Welfare Centre for Epidemiology. The data from Statistics Sweden consisted of 473,800 foreign-born individuals and 453,282 Swedish "controls".

It was not possible to find native-born matched Swedish controls for 20,518 of the foreign-born subjects. The number of matching criteria has thus been a limiting factor, but the matching has also lent strength to the comparisons made between foreign-born individuals and native Swedes, enabling us to control not only for age and gender but also for occupation and geographical area of living.

Thus, 97,367 individuals in total (13.2%) were excluded from the original database. Exclusion criteria were lack of information or if a person had emigrated from Sweden. Those who had died before 1987 were also excluded, since the Swedish national register of hospital care and diagnosis (according to the International Classification of Diseases (ICD) system) covers the period 1987 and onwards. Individuals with hip fracture events were identified by the ICD codes S720-S722 and 820 [19,20] ensuring that no other osteoporotic fracture types were included.

The study was based on a large sample of 628 581 individuals, 321,407 of whom were foreign-born and 307,174 of whom were Swedish-born "controls".

Hip fracture were registered according to the system of International Classification of Diseases (ICD) revision 9 (1997) or 10 (1998).

Classification of hip fracture was not dependent on the individual physician and his/her transformation of facts, symptoms, and signs into a specific diagnosis and it is most unlikely that differences should not occur between different physicians and between different hospitals. Since hip fracture requires in-hospital care, only few cases might have been missed during the study period since the inpatient registry is complete and covers all hospitals in Sweden.

A limitation of the study was, however, that information on some well-known risk factors for hip fractures, such as smoking, alcohol consumption, dietary habits, and BMI, was not available.

The database reflects the migrant population in Sweden in 1970. Further studies on the current population Swedish are required, since the migration pattern has changed from a large proportion of labour migrants to a more mixed population, with more migrants coming from non-European countries as refugees or asylum seekers the past decades.

The data used to establish the database originated from the Population and Housing Census of 1970, which is considered to be a total census, and it was compulsory to take part. The number of dropouts has not been estimated for the total census, only for some of the variables such as "occupation", and Statistics Sweden estimates that the dropout rate on this variable was 3.5-4.5%. It can only be speculated whether non-participation in the census was related to health problems, and whether there were a number of migrants who did not take part (as well as a number of Swedes). Other reasons for migrants not participating in the census could be language problems.

## Competing interests

The authors declare that they have no competing interests

## Authors' contributions

BA, KH and SE conceived the project. BA made the statistical analysis and drafted the manuscript. All authors contributed to the study design, critically revised and approved of the final manuscript.

## Pre-publication history

The pre-publication history for this paper can be accessed here:

http://www.biomedcentral.com/1471-2474/11/203/prepub
